# Mucins in neoplastic spectrum of colorectal polyps: can they provide predictions?

**DOI:** 10.1186/1471-2407-10-537

**Published:** 2010-10-07

**Authors:** Mahsa Molaei, Babak Khoshkrood Mansoori, Reza Mashayekhi, Mohsen Vahedi, Mohamad Amin Pourhoseingholi, Seyed Reza Fatemi, Mohammad Reza Zali

**Affiliations:** 1 Research Institute for Gastroenterology and Liver Diseases, Taleghani Hospital, Shahid Beheshti University, M.C., Tehran, Iran

## Abstract

**Background:**

The significance of expression of different mucins in succession of malignant transformation of colorectal polyps is not determined yet. The aim of the present study was to determine the pattern of expression of MUC1, MUC2, MUC5AC and MUC6 in colorectal polyps and to evaluate the applicability of using mucin expression in predicting the extent of malignant transformation in colorectal polyps.

**Methods:**

A total of 454 polyp specimens comprising 36 hyperplastic polyps, 15 serrated adenomas, 258 tubular adenomas, 114 tubulovillous adenomas, and 31 villous adenomas were included in this study, and were immunostained for MUC1, MUC2, MUC5AC and MUC6 by using mucin specific antibodies.

**Results:**

MUC1 and MUC6 were absent in all hyperplastic polyps and their expression was higher in serrated and traditional adenomas. Only 5 cases including 2 serrated adenomas, 1 tubulovillous adenoma, and 2 villous adenomas stained negative for MUC2. The highest expression of MUC5AC was observed in serrated adenomas followed by tubular adenomas. Binary logistic regression analysis indicated that positive staining for MUC1, and MUC6, and negative staining for MUC2 would increase the risk of invasion to mucosa or the muscularis mucosae in colorectal polyps. Ordinal regression analysis demonstrated a positive association between the level of staining for MUC1 and risk of being of high configuration/grade in colorectal polyps.

**Conclusions:**

MUC1, MUC2, MUC5AC, and MUC6 have the potential to be used as predictors of malignant transformation and invasion to mucosa or the muscularis mucosae in colorectal polyps. The most reliable predictions can be achieved by determining the level of expression of MUC1.

## Background

Mucins are high molecular weight glycoproteins that are produced, stored and secreted by mucosal epithelial cells [1], and act as the main part of the mucus protecting layer of the gastrointestinal tract. The protective role of mucins is achieved by the presence of oxygen bonds that attach the long sugar side-chains of the mucins to tandem repeat protein structures that are rich with serine and threonine amino acids [2]. These bonds hinder the mucin degradation by inhibition of protease activity, and so preserve the viscosity and density of mucins [2].

Various mucin proteins are encoded by different MUC genes [3-9]. MUC1 gene is located on chromosome 1q21-24 and codes for a transmembrane mucin, which is rarely expressed in normal colorectal mucosa [3]. MUC2 gene, which is aboundantly expressed in colorectal goblet cells, encodes a secretory glycoprotein [4]. The secretory MUC3 gene product is normally expressed in colorectal mucosa [4,5]. MUC4 gene encodes a transmembrane glycoprotein. This gene is located on chromosome 3q29 and predominates in intestinal mucosa [6]. MUC5AC gene forms a cluster on chromosome 11p15.5 with MUC2 gene, and codes for a secretory mucin in stomach [7]. Similarly, MUC6 gene is typically expressed in stomach [5].

A growing body of evidence advocates the idea that expression of MUC genes and distribution of their products alter dramatically in certain types of colorectal polyps and neoplasms [2,10]. These alterations take place through several mechanisms that include aberrant glycosylation of mucin side chains, immunoreactivity of mucin core peptide, deletion of normally expressed antigens, expression of blood-group incompatible antigens, and de novo appearance of new antigens [2]. The secretion of MUC2 gene products, for example, is suppressed in non-mucinous colorectal neoplasms [4,11], or MUC5AC and MUC6, which are normally absent in colorerectal mucosa, are expressed de novo in villous and tubulovillous colorectal adenomas [12,13]. MUC1 is also believed to become upregulated in colorectal cancers (3).

The current consensus classifies majority of the colorectal polypoid lesions into three major levels of malignant transformation: hyperplastic polyps (HPPs), serrated adenomas, and conventional (traditional) adenomas [14-16]. It is not still elucidated, however, which mucin alterations at which stage of malignant transformation take place or become detectable [17]. For instance, there is a large body of controversy regarding the alterations of MUC5AC and MUC6 in colorectal polyps. While these two mucins were reported previously to be more expressed in medium size/stage adenomas [12], more recent data reports MUC5AC to be involved in early stages of malignant transformation, and MUC6 in final stages [18].

The main objective of this study was to determine the extent of expression of MUC1, MUC2, MUC5AC, and MUC6 in different levels of neoplastic transformation of colorectal mucosa according to immunohistochemistry results. Moreover, we aimed at determining the predictive value of mucin expression on the malignant transformation of colorectal polyps.

## Methods

### Patients

In the present study, polyp specimens of all cases of colorectal polyp, who had undergone colonoscopy and biopsy from 2005 to 2008, were retrospectively studied. Clinical and demographic data were retrieved from the available hospital records of Taleghani Hospital, Shahid Beheshti University, M.C. Colorectal polyps were classified according to their location as right sided (proximal to splenic flexure), left sided (distal to splenic flexure), and rectal.

Hematoxylin and Eosin (H&E) slides and Formalin-fixed, paraffin-embedded tissue polyp specimens including 36 hyperplastic polyps, 15 serrated adenomas, 258 tubular adenomas, 114 tubulovillous adenomas, and 31 villous adenomas were obtained from the archives of pathology department of Taleghani Hospital, Shahid Beheshti University, M.C. This classification of polyps will be referred to as polyp configuration hereafter.

Available H&E slides were examined initially by two pathologists to recheck the original histological diagnosis of specimens. Traditional adenomas were classified in advance into three categories of mixed adenomatous-hyperplastic, Low-grade adenomatous, and High-grade adenomatous polyps according to grade of glandular dysplasia.

### Immunohistochemistry

Tissue samples were sectioned (4 μm), deparaffinized in xylene, and rehydrated in descending alcohol gradient. Blocking solution was used to block the endogenous peroxidase activity. For antigen retrieval, sections were treated in advance in boiling citrate buffer (ph 6.0) in microwave oven. Sections were immunostained and incubated afterwards with primary antibodies for MUC1 (Invitrogen, Clone:VU-4-H5), MUC2 (Invitrogen, Clone:CCP58), MUC5AC (Invitrogen, Clone:45M1), and MUC6 (Invitrogen, Clone:1G8). Then, slides were treated with Envision (DAKO, REAL Envision) for 20 minutes. To visualize immunoreactivity, diaminobenzidine was used and samples were counterstained with hematoxylin. Ascending alcohol gradient was used eventually to dehydrate the sections.

Specimens were observed by two pathologists. The level of staining for each mucin was calculated by determining the percentage of immunostained cells per high power field, and reported as 0 (no staining), 1+ (up to 30%), 2+ (30-60% of cells stained), 3+ (more than 60% of cells stained). Clinical and demographic data were compared according to presence (positiveness) or absence (negativity) of immunohistochemical staining for each mucin.

### Statistical analysis

Differences of distribution between the categorical variables were examined with chi-square test and Fisher's exact test in case of need. For quantitative variables Student's t-test was employed. Three factors of invasion status (to mucosa or muscularis mucosae and not beyond), configuration, and grading of polyps were considered as indicators of malignant transformation of polyps. Binary logistic regression analysis, with covariates of gender, age, polyp site, and MUC status (+/-) included, was performed to estimate the risk of invasion (as defined earlier) according to mentioned factors. In case of polyp configuration and grading, ordinal regression analysis was employed, with factors of expression level of different mucins, tumor site, and gender included, in order to generate predictions and evaluate the importance of mentioned predictor variables. Age was not included in this model because as a continous variable it would decrease the reliability of the model. Reported P values of less than 0.05 were considered to represent the statistical significance.

### Ethical considerations

The present study was undertaken with approval and under direct supervision of ethics committee of Research Institute for Gastroenterology and Liver Diseases, affiliated to Shahid Beheshti University, M.C.

## Results

The study population comprised 454 patients with mean age of 59.5 ± 14.61 (range 15-89), and male to female ratio of 1.48. 34% of polyps were of right colon origin, 50% of left colon, and 16% of rectum. 36 polyps (7.9%) were hypersplastic, 15 (3.3%) serrated adenoma, and 403 (88.8%) traditional adenoma. Traditional adenomas included 258 tubular adenoma specimens, 114 tubulovillous adenomas, and 31 villous adenomas. Invasion (just to mucosa and muscularis mucosae) was observed only in traditional adenomas, especially the villous type (Table 1).

**Table 1:** invasion^1 ^status, grading, and site of traditional adenomas

	**TA**^**2**^	**TVA**^**2**^	**VA**^**2**^	*P *value
Invasion	3 (1.2%)	10 (8.8%)	11 (35.5%)	< 0.0001
Grade				< 0.0001
Mixed adenomatous-hyperplastic	17 (6.6%)	0%	0%	
Low-grade adenomatous	217 (84.1%)	76 (66.7%)	16 (51.6%)	
High-grade adenomatous	24 (9.3%)	38 (33.3%)	15 (48.4%)	
Polyp Site				< 0.0001
Right colon	103 (39.9%)	27 (23.7%)	5 (16.1%)	
Left colon	118 (45.7%)	64 (56.1%)	21 (67.7%)	
Rectum	37 (14.3%)	22 (19.3%)	5 (16.1%)	

**^1 ^**Invasion not beyond mucosa or muscularis mucosae; ^2 ^Abbreviations: TA, tubular adenoma; TVA, tubulovillous adenoma; VA, villous adenoma

### IHC results for MUC1, MUC2, MUC5AC, and MUC6

MUC1 was positive in 106 (23.3%) polyps, of which none was hyperplastic. The expression of MUC1 was significantly higher in traditional adenomas than serrated adenomas/hyperplastic polyps (23.5% vs. 7.8%, P = 0.005). The main difference between the MUC1 positive and negative polyps was in the level of neoplastic transformation (Table 2). Higher levels of MUC1 expression was also observed among traditional adenomas especially tubulovillous and villous adenomas (Table 3). Immunostaining for MUC2 was positive in 449 cases (98.9%). Only five cases had negative MUC2 staining, of which 2 were serrated adenomas, 1 was tubulovillous adenoma, and 2 were villous adenomas (Table 2). In comparison to hyperplastic polyps, traditional adenomas showed lower levels of MUC2 expression (Table 3).

**Table 2:** Clinical and demographic differences of polyps with and without MUC immunoreactivity

	MUC1 Staining		MUC2 Staining		MUC5AC Staining		MUC6 Staining	
	
	+	-	+	-	+	-	+	-
Age								
Mean	59.86	59.45	59.55	59.00	59.37	59.67	61.14	59.10
	*P = 0.803*		*P = 0.933*		*P = 0.837*		*P = 0.231*	
Gender								
Male	72	198	266	4	112	158	61	209
Female	33	150	182	1	83	100	39	144
	*P = 0.020*		*P = 0.462*		*P = 0.491*		*P = 0.824*	
Site								
Right colon	37	119	154	2	73	83	27	127
Left colon	54	172	224	2	94	132	57	169
Rectum	15	56	70	1	27	44	14	57
	*P = 0.908*		*P = 0.975*		*P = 0.370*		0.404	
Configuration								
Hyperplastic	0	36	36	0	13	23	0	36
Serrated	4	11	13	2	10	5	4	11
TA**^1^**	69	189	258	0	117	141	57	201
TVA**^1^**	21	93	113	1	43	71	28	86
VA**^1^**	12	19	29	2	12	19	11	20
	*P = 0.001*		*P < 0.0001*		*P = 0.182*		*P = 0.007*	
Type								
Hyperplastic	0	36	36	0	13	23	0	36
Serrated	4	11	13	2	10	5	4	11
Mixed adenomatous-hyperplastic	0	17	17	0	3	14	3	14
Low-grade adenomatous	70	239	309	0	136	173	74	235
High-grade adenomatous	32	45	74	3	33	44	19	58
	*p < 0.0001*		*p < 0.0001*		*P = 0.069*		*P = 0.021*	
Invasion**^2^** % within MUC+/-	9.4%	4%	4.7%	60%	3.6%	6.6%	13%	3.1%
	*P = 0.029*		*p < 0.0001*		*P = 0.161*		*p < 0.0001*	

**^1 ^**Abbreviations: TA, tubular adenoma; TVA, tubulovillous adenoma; VA, villous adenomas; **^2 ^**Invasion not beyond mucosa or muscularis mucosae

**Table 3:** Level of expression of different mucins in colorectal polyps

	**HPP**^**1**^	**SA**^**1**^	**TA**^**1**^	**TVA**^**1**^	**VA**^**1**^	*P *value
MUC1 expression						
0	36	11	189	93	19	< 0.000
1+	0	3	58	13	2	
2+	0	1	11	8	8	
3+	0	0	0	0	2	
MUC2 expression						
0	0	2	0	1	2	< 0.000
1+	3	2	32	16	2	
2+	22	6	145	69	15	
3+	11	5	81	28	12	
MUC5AC expression						
0	23	5	141	71	19	< 0.000
1+	10	2	16	13	10	
2+	2	6	74	27	2	
3+	1	2	27	3	0	
MUC6 expression						
0	36	11	201	86	20	0.067
1+	0	3	48	23	8	
2+	0	1	8	5	2	
3+	0	0	1	0	1	

**^1 ^**Abbreviations: HPP, hyperplastic polyps; SA, serrated adenoma; TA, tubular adenoma; TVA, tubulovillous adenoma; VA, villous adenoma

MUC5AC was positive in 195 specimens. We could find no significant difference in distribution of MUC5AC in different polyps. In general, majority of colorectal polyps in this study had negative expression of MUC5AC. The exception, however, were serrated adenomas of which 66% were MUC5AC positive (Table 2). More than 50% of serrated adenomas had MUC5AC expression level of 2+ or more (Table 3). In addition, meanwhile MUC6 was positive in about one third of tubulovillous adenoma and villous adenomas, it was negative in all hyperplastic polyps (Table 2). Nonetheless, no statistical significant could be found for difference of MUC6 expression level in various polyp types (Table 3). Moreover, we could find no association between expression of MUC6 with MUC5AC; in other words, only 24.1% of MUC5AC positive specimens were also positive more MUC6, and 47% of MUC6 positive specimens, had also positive immunostaining for MUC5AC (P = 0.354)

Certain patterns of MUC expression were significantly associated with invasion of polyps to mucosa or muscularis mucosae, including the up-regulation of MUC1 (marginal significance), absence of MUC2, and positive MUC6 expression (Table 2). Regression analysis, with covariates of gender, age, polyp site, and MUC status included, indicated that negative staining for MUC2, Positive staining for MUC6, and left-sidedness would increase the risk of mucosal or muscularis mucosae invasion in colorectal polyps significantly (Table 4).

**Table 4:** Regression analysis determining the risk of invasion^1 ^in colorectal polyps

	*P *value	Odds Ratio	95% confidence interval	
			
			Lower bound	Upper bound
MUC1, negative vs. positive	0.069	0.407	0.155	1.071
MUC2, negative vs. positive	0.002	29.729	3.424	258.140
MUC5AC, negative vs. positive	0.132	2.142	0.794	5.776
MUC6, negative vs. positive	0.001	0.203	0.081	0.506
Gender, female vs. male	0.053	2.503	0.988	6.337
Polyp site, left colon and rectum vs. right colon	0.049	3.834	1.005	1.005
Age**^2^**,	0.335	0.985	0.956	1.015

**^1 ^**Invasion to mucosa or muscularis mucosae and not beyond; **^2^**age was entered as continuous variable

The ordinal regression analysis indicated that the described model (see statistical analysis), would give adequate predictions either in case of configuration and grading (P < 0.001, Chi square = 65.32 and 64.99 respectively). The model with the complementary log-log link (table 5) shows that the four thresholds of the model equation were significantly different from zero and substantially contributed to the values of the response probability in different configurations. Moreover, the configuration of polyps was significantly associated with three covariates: polyp site, and level of expression of MUC1 and MUC5AC (table 5).

**Table 5:** ordinal regression determining the risk of ranking higher in the configuration of colorectal polyps

	*P *value	Regression Coefficient	95% confidence interval	
			
			Lower bound	Upper bound
Threshold				
HPP**^1^**	< 0.001	-20.295	-22.239	-18.325
SA**^1^**	< 0.001	-19.929	-21.864	-17.993
TA**^1^**	< 0.001	-17.580	-19.508	-15.658
TVA**^1^**	< 0.001	-16.586	-18.506	-14.666
MUC1				
0	< 0.001	-17.609	-18.075	-17.142
1+	< 0.001	-17.810	-18.334	-17.286
2+	-	-16.873	-16.873	-16.873
MUC5AC				
0	0.008	0.583	0.152	1.014
1+	0.001	0.865	0.345	1.384
2+	0.039	0.480	0.024	0.937
Polyp site,				
left colon and rectum	< 0.001	0.417	0.194	0.641
right colon				

**^1 ^**Abbreviations: HPP, hyperplastic polyps; SA, serrated adenoma; TA, tubular adenoma; TVA, tubulovillous adenoma

Using the model with the negative log-log link to build the ordinal regression, the grade of glandular dysplasia was found to be significantly associated with the three variables of expression levels of MUC1 and MUC2, and polyp site (table 6).

**Table 6:** ordinal regression determining the risk of being of higher grades

	*P *value	Regression Coefficient	95% confidence interval	
			
			Lower bound	Upper bound
Threshold				
HPP**^1^**	< 0.001	-8.692	-10.818	-6.566
SA**^1^**	< 0.001	-8.515	-10.640	-6.391
Mixed adenomatous-hyperplastic	< 0.001	-8.352	-10.467	-6.228
Low-grade adenomatous	< 0.001	-5.763	-7.855	-3.670
MUC1				
0	< 0.001	-6.857	-7.451	-6.263
1+	< 0.001	-6.172	-6.807	-5.537
2+	-	-5.189	-5.189	-5.189
MUC2				
0	0.043	-1.345	-2.651	-0.040
1+	0.251	.221	-0.156	0.598
2+	0.682	.052	-0.196	0.299
Polyp site,				
Left colon and rectum	0.047	0.237	0.003	0.470
Right colon				

**^1 ^**Abbreviations: HPP, hyperplastic polyps; SA, serrated adenoma

## Discussion

MUC1 and MUC2 normally belong to colorectal mucosa; however, while the expression of MUC1 is a rare occasion in normal colorectal tissue, MUC2 is the predominant secretory colorectal mucin [17-21]. MUC5AC and MUC6, which are gastric mucins, are believed to express de novo in certain colorectal polyps [12-14].

The pattern of distribution of different mucins described in this study is in general in accordance with findings of previous studies in this regard [2,4,12,14,16-21]. While the highest expression of MUC1, and MUC6 was observed in villous adenomas, they were totally absent in hyperplastic polyps. MUC2, in contrast, was completely expressed in hyperplastic polyps and tubular adenomas, but was absent in some serrated adenoma, tubulovillous adenoma, and villous adenoma specimens.

Results of MUC5AC, however, were in favor of previous studies depicting a higher expression of MUC5AC in middle stages of malignant transformation [12]. Positive coefficients for MUC5AC in ordinal regression analysis (table 5) indicate that increase of MUC5AC expression level does not increase the likelihood of a polyp to be of villous adenoma configuration. These results were consistent with that of table 3, in which expression of MUC5AC was more prominent in tubular adenoma, and to a lesser extent in serrated and tubulovillous adenomas.

As is shown in table 2, when it comes to the distribution pattern of different mucins, serrated adenomas act as if they rank higher in the sequence of grading and configuration. This is much more tangible in case of MUC2 that was absent in 13.3% of serrated adenomas, and MUC5AC that was present in 66.6% of these cases. These findings advocate the results reported by some previous studies regarding the high abnormality of mucins expression in serrated adenomas [14,18].

Along with determining the pattern of distribution of mucins in colorectal polyps, the present study benefits from the added value of ordinal regression analysis as part of its methodology. The model fitting information tables indicated that our models would give better predictions than intercept-only models (see results) [22-24]. Moreover, the Pearson chi-square and deviance chi-square were non-significant for both models, implying the well fitting. As no scale component was included, test of parallel lines assumption was applicable [22], and yielded significance of less than 0.001 for both models (not presented in results section).

Given the regression analyses, (binary logistic and ordinal) it is implied that different mucins can provide certain predictions regarding mucosal and muscularis mucosae invasion, grading and configuration of colorectal polyps. In this respect, MUC 2 and 6 were shown to be associated with invasive behaviour of these polyps. Level of expression of MUC1 and 5 was associated with the level of configuration, and level of expression of MUC 1 and 2 could provide predictions on grading of colorectal polyps.

MUC1 exhibited negative regression coefficients in both of these ordinal regression models, indicating that polyps that expressed lower levels of immunostaining for MUC1 were unlikely to rank higher in polyp configuration/grading. Moreover, in binary logistic regression analysis, a marginally significant association was observed between the expression of MUC1 and invasion risk. At the present state of knowledge, these findings do not imply that MUC1 can be used as a tumor marker. But based upon these models, one can confidently argue about the potential applicability of MUC1 expression as a predictor of malignant transformation, and invasive behavior of colorectal polyps. Negative staining for MUC2 was shown in the binary logistic regression model to be valuable in predicting the risk of mucosal and muscularis mucosae invasion in colorectal polyps. In addition, in ordinal regression analysis, it was shown to reduce the likelihood of a polyp to be of high grades. Positive expression of MUC6 increased the risk of invasion (as defined earlier) significantly; however, no predictions could be elicited in terms of grading and configuration by using MUC6.

Applicability of mucins in predicting certain characteristics of colorectal polyps (invasion, configuration and grading) evokes the notion that maybe mucins expression could be employed in predicting the extent of malignant transformation in colorectal polyps. Future studies, however, would prove or disprove this hypothetical potential.

In case of polyp site, significant associations were elicited in all of regression models with invasion, grading and configuration. Given these analyses, beside the expression status of MUC1, left sidedness of colorectal polyps corresponded to increased risk of invasion to mucosa and muscularis mucosae, having high configuration and polyp grading.

Our models had their own limitations too. The major limitation, which arised from the retrospective nature of the study, was that data of normal colorectal mucosa were not included in the statistical models. The fact is that the pattern of expression of MUC1, MUC2, and MUC6 that is reported in the present article for hyperplastic polyps, is identical to what is reported in other studies for normal mucosa [14,17,18]. However, this does not hold true for MUC5AC, which indicates that lack of inclusion of normal mucosa data could have influenced the regression results of MUC5AC to a larger extent.

The other limitation, arised from the recent concept that serrated adenomas might go through a different pathway of malignant transformation [25-28]. Majority of colorectal polyps can be classified as hyperplastic polyps, serrated adenomas and traditional adenomas [2]. Owning mixed features of hyperplastic polyps and traditional adenomas, serrated adenomas were quite recently introduced as a distinct entity that lies between hyperplastic polyps and traditional adenomas in the sequence of malignant transformation of colorectal polyps [29]. However, more recent data indicate that serrated adenomas can get involved in an alternative carcinogenesis pathway that does not include traditional adenomas [25-28]. This could cast a shadow of doubt on the presumed ordinal hierarchy towards the malignancy that was considered in this study in terms of grading and configuration. Nonetheless, at the present state of knowledge, we preferred to adhere to these two commonly used classifications, and not to exclude serrated adenomas.

## Conclusions

To conclude, we think that mucins have the potential provide predictions on invasive behaviour, grading and configuration of colorectal polyps. But the predictive value of various mucins differ based upon what is going to be predicted. Almost all of mucins that are studied here can provide predictions on the invasive behavior of colorectal polyps; however, only MUC1 can provide such information in respect to both the grading and configuration of colorectal polyps. Thus, MUC1 is the most reliable predictor of malignant transformation of colorectal polyps among the mucins that are studies in this article.

## Competing interests

The authors declare that they have no competing interests.

## Authors' contributions

MM participated in study design, carried out the immunostaining, and was one of the blinded pathologists who examined the slides. BKM participated in the study design, coded the slides (blinding), performed the statistical analysis, and wrote the article. RM participated in immunostaining, and was one of blinded pathologists who examined the slides. MV and MAP participated in data collection, mining, and statistical analysis. SRF participated in study design, provided data, and funding by RIGLD.MRZ participated in study design, and supervised the study. All authors read and approved the final manuscript.

## Pre-publication history

The pre-publication history for this paper can be accessed here:

http://www.biomedcentral.com/1471-2407/10/537/prepub
